# Phenotypic Effects of Homeodomain-Interacting Protein Kinase 2 Deletion in Mice

**DOI:** 10.3390/ijms22158294

**Published:** 2021-08-02

**Authors:** Davide De Biase, Valeria Valente, Andrea Conte, Francesca Cammarota, Nicola Boccella, Lucia D’Esposito, Ilaria d’Aquino, Orlando Paciello, Simona Paladino, Giovanna Maria Pierantoni

**Affiliations:** 1 Department of Veterinary Medicine and Animal Productions, University of Naples Federico II, 80137 Naples, Italy; davide.debiase@unina.it (D.D.B.); ilaria.daquino@unina.it (I.d.); paciello@unina.it (O.P.); 2 Department of Molecular Medicine and Medical Biotechnology, University of Naples Federico II, 80131 Naples, Italy; valeria.valente2@unina.it (V.V.); andrea.conte@nih.gov (A.C.); franc.cammarota@studenti.unina.it (F.C.); 3 Department of Advanced Biomedical Sciences, University of Naples Federico II, 80131 Naples, Italy; nicolaboccella@live.it; 4 Centro Servizi Veterinari, University of Naples Federico II, 80131 Naples, Italy; lucia.desposito@unina.it

**Keywords:** HIPK2, myopathic changes, knock-out mice

## Abstract

Homeodomain-interacting protein kinase 2 (HIPK2) is a serine-threonine kinase that phosphorylates various transcriptional and chromatin regulators, thus modulating numerous important cellular processes, such as proliferation, apoptosis, DNA damage response, and oxidative stress. The role of HIPK2 in the pathogenesis of cancer and fibrosis is well established, and evidence of its involvement in the homeostasis of multiple organs has been recently emerging. We have previously demonstrated that *Hipk2*-null (*Hipk2*-KO) mice present cerebellar alterations associated with psychomotor abnormalities and that the double ablation of HIPK2 and its interactor HMGA1 causes perinatal death due to respiratory failure. To identify other alterations caused by the loss of HIPK2, we performed a systematic morphological analysis of *Hipk2*-KO mice. Post-mortem examinations and histological analysis revealed that *Hipk2* ablation causes neuronal loss, neuronal morphological alterations, and satellitosis throughout the whole central nervous system (CNS); a myopathic phenotype characterized by variable fiber size, mitochondrial proliferation, sarcoplasmic inclusions, morphological alterations at neuromuscular junctions; and a cardiac phenotype characterized by fibrosis and cardiomyocyte hypertrophy. These data demonstrate the importance of HIPK2 in the physiology of skeletal and cardiac muscles and of different parts of the CNS, thus suggesting its potential relevance for different new aspects of human pathology.

## 1. Introduction

Homeodomain-interacting protein kinase 2 (HIPK2) is a member of a protein family that includes four nuclear serine-threonine kinases (HIPK1, HIPK2, HIPK3, and HIPK4) [1]. Originally identified as corepressors of homeodomain transcription factors, HIPKs are able to phosphorylate and modulate the activity of various transcriptional regulators and chromatin modifiers, thus playing an important role in embryonic development, and in a multitude of cellular processes [2]. HIPKs also act as transcriptional coregulators in important signal transduction pathways, such as Wnt/β-catenin, TGF-β, MAPK, Notch, Salvador–Warts–Hippo, and androgen receptor (AR), contributing to their cross-talk [3,4]. HIPK2 is the best characterized member of the family and is actively involved in the regulation of cell proliferation, apoptosis, DNA damage response, cytokinesis, transcription, and protein stability [5,6,7,8,9,10,11]. HIPK2 expression and activity are tightly regulated by post-translational modifications and miRNAs, and its functions strongly depend on the cellular context, and on its subcellular localization, which can be nuclear and/or cytoplasmic [8,9,10].

Because of their important role in the regulation of cell proliferation and survival, HIPK proteins have traditionally been linked to the pathogenesis of cancer and fibrosis, which are often associated with deregulated activity or expression of HIPKs [12]. In particular, HIPK2 is considered a bona fide tumor suppressor, primarily because of its involvement in DNA damage repair, induction of apoptosis, and regulation of cell proliferation [4,5,6,7,8,9,10,11,12,13]. Indeed, *Hipk2^−/−^* mice are more susceptible to skin chemical carcinogenesis [11], and HIPK2 expression is down-regulated in breast, thyroid, and colon carcinomas [14,15]. HIPK2 is also involved in signaling pathways crucial for the induction of kidney and lung fibrosis [12].

The relevant physiological role of HIPK2 emerged also from the phenotype of *Hipk2*-null (*Hipk2*-KO) mice. *Hipk2* genetic ablation affects the mouse body size, being *Hipk2*-KO mice significantly smaller than their wild-type littermates, as well as the proliferation of different cell types, including fetal liver cells [16], mouse embryo fibroblasts (MEFs) [17,18], bone marrow [19], and sensory neurons [20]. Moreover, *Hipk2*-KO mice show several neuronal defects, including a reduction of midbrain dopamine neuron survival [21] and apoptosis of cerebellar Purkinje cells, associated with several psychomotor behavioral abnormalities [22].

Recent findings suggest that HIPK2 may be important also for the biology of cardiac and skeletal muscle cells. In particular, the involvement of HIPK2 in heart pathophysiology is suggested by the evidence that a reduction of HIPK2 in cardiomyocytes leads to cardiac dysfunction in mice and that cardiac HIPK2 expression is significantly reduced in human end-stage ischemic cardiomyopathy, in comparison with non-failing myocardium [23]. On the other hand, HIPK2 expression strongly increases after skeletal muscle contusion in neutrophils, macrophages, and myofibroblasts [24]. Finally, we recently reported that the double KO of HIPK2 and high-mobility group A1 (HMGA1), a chromatin non-histone protein previously identified as HIPK2 interactor and substrate, causes perinatal death due to respiratory failure, associated with impaired lung development and reduction in surfactant proteins, as well as reduced expression of thyroid differentiation markers [25].

All these data suggest a pleiotropic involvement of HIPK2 in the physiological homeostasis of different organs and tissues and, potentially, in the pathogenesis of multiple diseases. On this basis, to confirm the importance of HIPK2 in the above-mentioned organs and tissues, and try to identify other organs or tissues whose homeostasis may depend on HIPK2, we performed a systematic morphological analysis of *Hipk2*-KO mice. Post-mortem examination and histological analysis revealed that *Hipk2* loss causes neuronal alterations throughout the central nervous system (CNS), a myopathic phenotype, and cardiac fibrosis associated with increased cardiomyocyte size.

## 2. Results

### 2.1. Hipk2-KO Mice Show Neuronal Alterations

**Figure 1:**
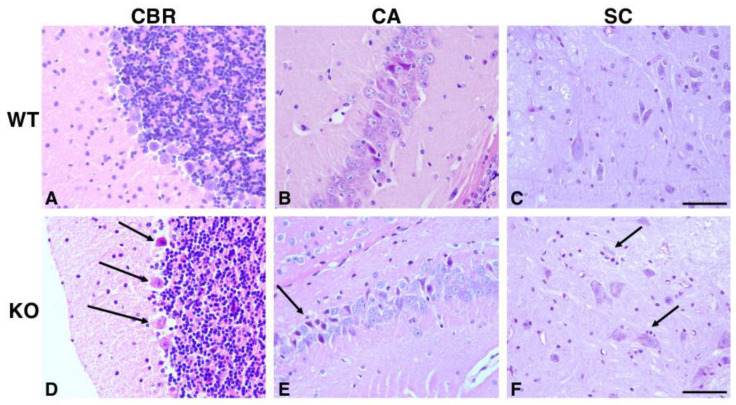
Histological analysis of the cerebellum (CBR), hippocampus (cornu ammonis) (CA), and spinal cord (SC) from 18-month-old wild-type (WT) and *Hipk2*-KO (KO) mice. Representative images of H&E staining of WT (**A**–**C**) and KO (**D**–**F**) CBR, CA, and SC are shown. The arrows indicate neuronal loss (**D**,**E**) and satellite cells (**F**). Original magnification, 40×. Scale bar = 50 μm.

To better define the role of *Hipk2* in vivo, we examined the consequences of the disruption of the *Hipk2* gene by performing a systematic morphological analysis of *Hipk2*-KO mice. Mice homozygous for the *Hipk2*-null mutation develop normally and are fertile and viable [17,22]. Therefore, we decided to perform a post-mortem examination of 4-, 12-, and 18-month-old mice. Histological analyses of serial sections from organs and tissues confirmed that their structure and development is overall normal as previously reported [22,25]. However, the brain and spinal cord of *Hipk2*-KO mice showed mild to moderate changes, such as scattered neuronal loss in the Purkinje layer of the cerebellum (CBR; Figure 1D) and in cornu ammonis of hippocampus (CA; Figure 1E). Occasionally, several neurons appeared shrunken, hypereosinophilic, and with pyknotic nuclei. Cerebellar white matter showed mild vacuolation of the neuropil, with a large number of clearly defined and empty vacuoles of varying size, and gliosis. In the ventral horns of the spinal cord (SC), many neurons appeared swollen, vacuolated or with dispersed Nissl substance (chromatolysis-degenerative change). Occasionally, such neurons were surrounded by satellite cells (Figure 1F).

Furthermore, as shown in Figure 1, *Hipk2*-KO mice showed the presence of scattered hypereosinophilic necrotic neurons throughout the central nervous system (CNS), sometimes associated with satellitosis. This phenotype is suggestive of prior neuronal necrosis and may be compatible with an end-stage cellular response to irreversible injury, such as ischemia or metabolic dysfunction. Noteworthy, the above-mentioned neuronal alterations appear in 18-month-old KO mice, while they are absent in 4-month-old mice (not shown), and barely noticeable in 12-month-old mice (not shown). The brain of age-matched wild-type (WT) mice did not show any significant or specific histological alterations (Figure 1A–C). The late onset of these alterations is consistent with our recent report about the involvement of *Hipk2* in nervous system physiology and, in particular, in cerebellar homeostasis. In fact, we had already found that *Hipk2*-KO mice present both morphological and functional cerebellar alterations that become more evident with aging [22].

### 2.2. Hipk2-KO Mice Show a Mild Myopathic Phenotype

**Figure 2:**
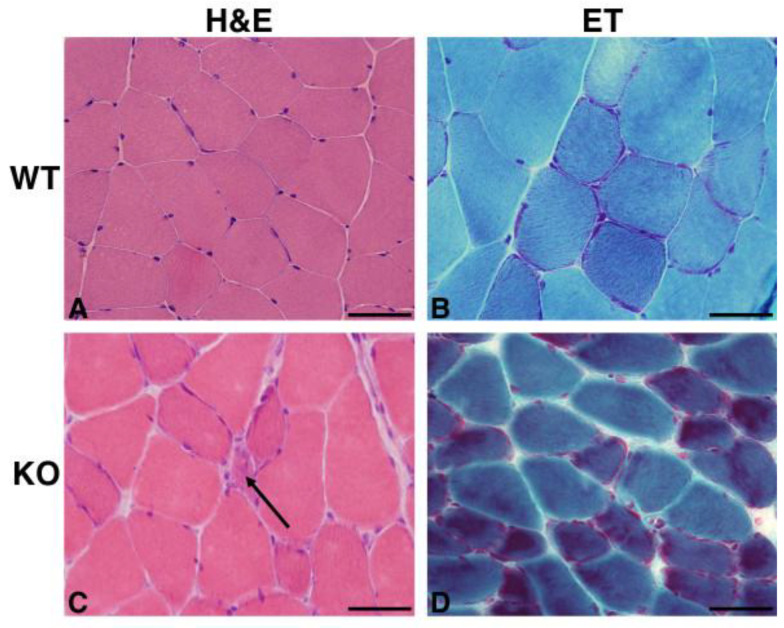
Morphological analysis of *Hipk2*-KO skeletal muscle. Representative H&E and ET staining of sections from skeletal muscle of 18-month-old WT (**A**,**B**) and *Hipk2*-KO (**C**,**D**) mice. The arrow indicates an atrophic angular fiber (**C**). Original magnification, 40×. Scale bar = 20 μm.

**Figure 3:**
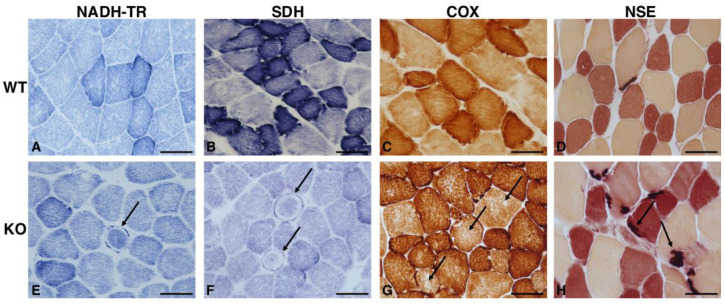
Characterization of *Hipk2*-KO skeletal muscle. Histochemical analysis of NADH-TR (**A**,**E**), SDH (**B**,**F**), COX (**C**,**G**), NSE (**D**,**H**) on sections of skeletal muscle of 18-month-old WT and *Hipk2*-KO mice are shown. Arrows in (**E**,**F**) indicate the presence of numerous ring fibers with subsarcolemmal positive deposits; arrows in (**G**) indicate negative cytoplasmic core; arrows in (**H**) indicate hypertrophic and fragmented neuromuscular junctions. Original magnification, 40×. Scale bar = 20 μm.

Since the cerebellar abnormalities of *Hipk2*-KO mice are associated with muscle and balance impairment, we hypothesized that morphological alterations may be present also at the muscular level. Consistently with our hypothesis, histological and histoenzymatic examination showed mild to moderate variability in fiber size, with the presence of both non-angular and angular atrophic fibers (Figure 2C) in 18-month-old KO mice, but not in the WT counterpart (Figure 2A). Occasionally, fibers with sarcoplasmic rimmed vacuoles, ring fibers and a mild to moderate increase in endomysial connective tissue were detected (Figure 2C,D). Engel’s trichrome (ET) staining showed the presence of subsarcolemmal red deposits (ragged red fibers) in approximately 10% to 30% of the fibers (Figure 2D), but no significant alteration of nerve branches was found. The skeletal muscle sections of age-matching WT mice presented a normal morphology (Figure 2B). Considering the importance of mitochondria in muscle cell metabolism, we evaluated the activity and distribution of these organelles in skeletal muscle cells by performing specific histoenzymatic stains, such as NADH-tetrazolium reductase (NADH-TR), succinate dehydrogenase (SDH), and cytochrome oxidase (COX). In *Hipk2*-KO mice, we found the presence of subsarcolemmal positive deposits in less than 10% of the fibers (Figure 3E,F), and negative cytoplasmic core in approximately 10% of the fibers (Figure 3G). Notably, in one male mouse, we also found several ring fibers that are suggestive of chronic myopathy (Figure 3E,F). Positive sarcoplasmic deposits, representative of mitochondria accumulation, were detected in approximately 10% of the fibers (Figure 3G). Moreover, non-specific esterase (NSE) staining was performed in order to evaluate neuromuscular junctions, lipofuscin deposits, inflammatory cells and denervated fibers. This staining revealed that neuromuscular junctions appeared moderately hypertrophic, bizarre in shape, and occasionally fragmented or moderately thicker than normal in *Hipk2*-KO mice (Figure 3H). WT mice did not show any relevant pathological changes, either in mitochondrial activity and distribution, or in shape and size of neuromuscular junctions (Figure 3A–D). Finally, ATPase and PAS staining did not reveal any pathological alterations in terms of glycogen accumulation or fiber type grouping (Supplementary Figure S1).

**Figure 4:**
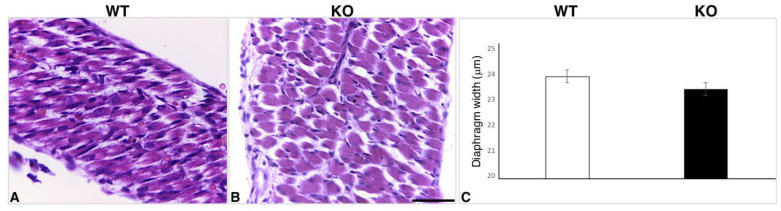
Histological analysis of embryonic skeletal muscle tissues in *Hipk2*-KO mice. Representative images of H&E-stained diaphragms from WT and *Hipk2*-KO embryos at 17.5 days post-coitum (dpc). (**A**,**B**) Histograms showing width of diaphragm of muscle cells in WT and *Hipk2*-KO embryos at 17.5 dpc. (**C**) Results are mean ± S.E.M. Original magnification, 40×. Scale bar = 50 μm.

**Figure 5:**
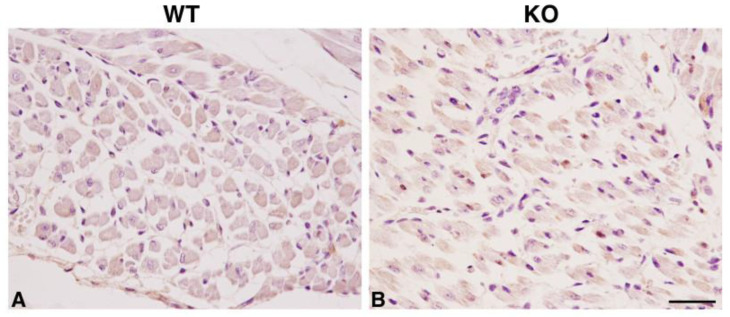
Analysis of skeletal muscle development in *Hipk2*-KO mice embryos. Immunohistochemical analysis of myogenin expression of skeletal muscle sections from WT (**A**) and *Hipk2*-KO (**B**) embryos at 17.5 dpc is shown. Original magnification, 40×. Scale bar = 50 μm.

To evaluate whether the muscle alterations caused by the lack of HIPK2 are already present during embryonic development, we performed a histological analysis of skeletal muscle tissues in E17.5 embryos. We found that the cell and tissue morphology of *Hipk2*-KO samples was not significantly different from that of WT ones (Figure 4A–C). In fact, we did not detect either a significant decrease in the number of muscle fibers or an alteration in their size. Consistently, the calculation of the relative cross-sectional area of individual muscle cells in the diaphragm revealed that *Hipk2*-KO muscle cells are not different from WT ones (Figure 4C). Furthermore, immunohistochemical analysis of the muscle development marker myogenin revealed a physiological granular, sarcoplasmic, and/or nuclear staining of skeletal muscle cells in both WT and *Hipk2*-KO embryos (Figure 5).

Altogether, these data revealed that HIPK2 is not required for skeletal muscle development in mice. Furthermore, adult *Hipk2*-KO mice present a mild myopathic phenotype characterized by moderate variability in fiber size, mitochondrial proliferation, sarcoplasmic inclusions, and morphological alterations of neuromuscular junctions.

### 2.3. Hearts of Hipk2-KO Mice Show Increased Cardiomyocyte Cell Size and Fibrosis

**Figure 6:**
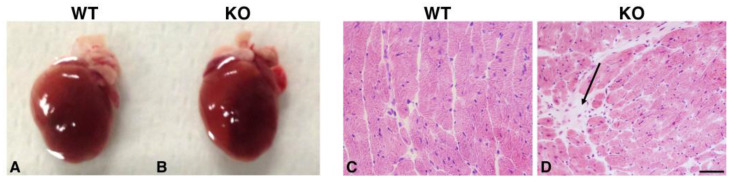
Analysis of the morphology of *Hipk2*-KO heart. Representative images of heart morphology (**A**,**B**) and H&E staining (**C**,**D**) from 18-month-old WT and *Hipk2*-KO mice are shown. The arrow in (**D**) indicates a fibrotic area. Original magnification, 40×. Scale bar = 20 μm.

**Figure 7:**
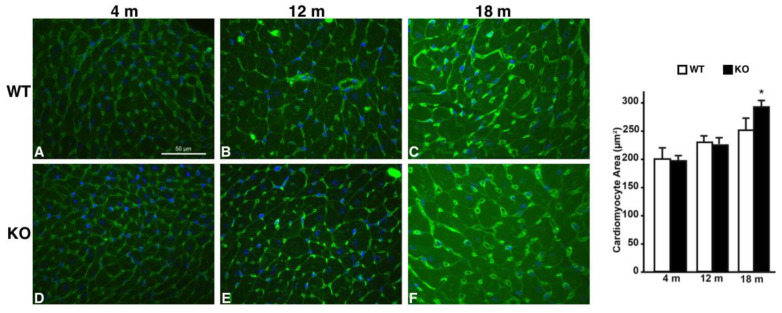
Effects of *Hipk2* deletion in cardiomyocyte cell size. Left panel: representative images of WGA staining of heart sections from WT (**A**–**C**) and *Hipk2*-KO (**D**–**F**) at 4, 12, and 18 months of age, respectively. Scale bar = 50 μm. Right panel: quantification of cross-sectional area of cardiomyocytes from WT and *Hipk2*-KO at 4, 12, and 18 months of age (* *p* < 0.05).

An important involvement of HIPK2 in cardiac pathophysiology has been recently reported [23]. To better define the potential morphological cardiac alterations consequent to *Hipk2* loss, we performed morphological cardiac analysis in our model. Consistently with what was reported by Guo et al. [23], despite the reduced dimension of the *Hipk2*-KO body size, we observed a marked increase in heart size in *Hipk2*-KO mice with respect to their WT counterpart (Figure 6A,B). Subsequently, we performed histological analysis on heart serial sections by H&E staining. As shown in Figure 6D, moderate fibrosis was detected in *Hipk2*-KO mice, whereas no significant alteration was revealed in WT mice (Figure 6C). Furthermore, to confirm the presence of cardiac functional abnormalities, we extracted the total RNA from WT and KO mice and performed a RT-qPCR analysis to evaluate the expression levels of specific heart failure markers such as atrial natriuretic peptide (NPPA), brain or B-type natriuretic peptide (NPPB), and myosin heavy chain 7 (MYH7). As expected, we found that the levels of these cardiac failure markers are increased in KO with respect to WT mice (Supplementary Figure S2). Moreover, the cross-sectional area of cardiomyocytes, measured by wheat germ agglutinin (WGA) staining, was significantly increased in Hipk2-KO (Figure 7F) compared to age-matched WT mice (Figure 7C), whereas no difference was detected in younger mice at 4 and 12 months of age (Figure 7A,B,D,E, respectively). Quantification of the cross-sectional area of cardiomyocytes is also reported (Figure 7, right panel). These data suggest that the loss of *Hipk2* induces an age-dependent cardiomyocyte hypertrophy in mice, which is in agreement with the long-term onset fibrosis displayed in these mice.

## 3. Discussion

The analysis of *Hipk2*-KO mice has been fundamental to shed light on the multiple functions that this kinase exerts in vivo. For instance, we reported that *Hipk2*-null mice present cerebellar abnormalities associated with muscle and balance impairment. In particular, at the morphological level, *Hipk2* genetic ablation induces a reduction of Purkinje neurons, consequent to apoptosis activation that causes atrophic cerebellar lobules and decreased cerebellar size [22]. In a previous report, the role of HIPK2 in the nervous system had already been suggested by the reduction of midbrain dopamine neuron survival observed in KO mice [21]. Recent papers also inferred the involvement of the kinase in muscle and cardiac pathophysiology. In fact, Wang et al. evaluated the effects of altered HIPK expression on the nervous system and muscles of *Drosophila melanogaster*, demonstrating that optimal levels of HIPKs are fundamental for the function of dopaminergic neurons and glial and muscle cells [26]. Moreover, the authors suggested that the manipulation of HIPKs may affect the structure and the function of the neuromuscular junctions [26]. Consistently, our results revealed that neuromuscular junctions in *Hipk2*-KO mice may show mild to moderate morphological changes, suggesting the importance of HIPKs in neuromuscular physiology and pathology.

On the other hand, Guo et al. reported that the lack of HIPK2 causes cardiac dysfunction in mice and that HIPK2 cardiac expression is reduced in human end-stage ischemic cardiomyopathy in comparison with non-failing myocardium [23]. Here we show that *Hipk2*-KO mice present increased cardiomyocyte cell size and fibrosis. Our findings about the cardiac role of HIPK2 consistently overlap with those of Guo et al., representing an important confirmation in an independently generated mouse model. 

Taken together, the data reported here provide more details about the role exerted by HIPK2 in CNS and cardiac physiology and show, for the first time, that the lack of the *Hipk2* gene causes a myopathic phenotype in mice. Consistently with what we observed in the cerebellum, and with other reports, most of the morphological abnormalities found in *Hipk2*-KO mice appear or become more evident in old individuals (18-month-old mice), suggesting that HIPK2 may be involved in the aging process of different organs. For example, despite the skeletal muscle phenotype exhibited by adult KO mice, when we compared WT and *Hipk2*-KO E17.5 embryos, we did not detect any significant difference in the morphology of skeletal muscle cells and tissues and in the expression pattern of the muscle development marker myogenin. Interestingly, HIPK2 is expressed during the latest phases of embryogenesis in several tissues, including skeletal muscle, and it has been identified to play a role in the kinetics of gene expression in proliferating myoblasts, and during the initial steps of myogenesis. According to this evidence, it would be reasonable to expect some muscular anomalies in *Hipk2*-KO embryos, which, instead, do not emerge from our analysis. Most probably, this is due to the fact that the closely related HIPK2 HIPK1 and/or HIPK3 paralogues may compensate for the lack of HIPK2 [14,27].

In general, the evidence that all the observed phenotypic features of the KO mice are relatively mild may be due to the functional redundancy of the different HIPK family members. In fact, *Hipk1* displays a very high homology degree to *Hipk2*, and the two genes play overlapping roles in mediating cell proliferation and apoptosis in response to morphogenetic and genotoxic signals during mouse development, as evidenced by embryonic lethality following their double KO [14]. The combination of *Hipk2* constitutive KO with an organ-specific *Hipk1*-KO (in the cerebellum, in the heart, or in muscle cells) may unleash much more severe phenotypes, revealing more information about the role and the functional interaction of these two HIPK proteins in vivo. Similarly, it is important to take into account that HIPK2 activity is strictly dependent on the functional interaction with its phosphorylation targets. For this reason, the ablation or mutation of one of these targets may contribute to causing a stronger phenotype. For example, we recently found that, when associated with the KO of its target *Hmga1*, the loss of *Hipk2* causes a lung and thyroid phenotype in the double KO mice, which is completely absent in the single KOs [25].

## 4. Materials and Methods

### 4.1. Animals and Ethics Statement

*Hipk2*-KO mice were generated in a mixed genetic background C57BL6/Sv129J and housed under identical conditions of temperature (21 ± 1 °C), humidity (60 ± 5%), and light/dark cycle, with free access to normal mouse chow [22,25]. To obtain WT and *Hipk2*-KO mice embryos, single male and female mice were time-mated and the females sacrificed under deep anesthesia at E17.5, and then embryos were collected and fixed in 4% paraformaldehyde. To assess the genotype of the mice, DNA was obtained from a small piece of tissue from the mouse. The tissue was incubated overnight at 60 °C with lysis buffer (50 mM Tris-HCl, 100 mM EDTA, 100 mM NaCl, 1% SDS, and 0.5 mg/mL proteinase K), and genomic DNA was extracted by adding 0.3 volumes of 6 M NaCl and precipitated with isopropyl alcohol. DNA concentration was determined using NanoDrop ND-1000 (NanoDrop, Wilmington, DE, USA), and an equal amount of DNA was used for PCR analysis. The annealing temperature for *Hipk2* alleles was 55 °C. The PCR products of *Hipk2* were separated on a 2% agarose gel, respectively. Gels were scanned with Chemidoc (Bio-Rad, Hercules, CA, USA). The following primers were used:

- Hipk2-Fw 5′-TAGTACCCAGGTGAACCTTGGAGT-3′
- Hipk2WT-Re 5′-GCTTCTCTCAAACTAAAGACCACGC-3′
- Hipk2KO-Re 5′-CAAAGGGTCTTTG

The experimental protocol was approved by the Animal Care Committee of the “Federico II” University of Naples, and in accordance with the principles and procedures outlined in the Guide for the Care and Use of Laboratory Animals published by the US National Institutes of Health (NIH Publication No. 85-23, revised 1996). For histological analysis, *n* = 10 animals/group were autopsied, and all tissues were examined regardless of their pathological status.

### 4.2. Morphology and Histochemistry

For each animal, a standardized base set of tissues was evaluated to identify the presence of relevant lesions [28]. Samples were collected and fixed in 10% buffered formalin for 48 h. E17.5 embryos were dissected from anesthetized females, and fixed in 4% paraformaldehyde overnight. Then, the samples were dehydrated, embedded in paraffin, and cut at 4 μm with a microtome.

Slides were stained with hematoxylin and eosin (H&E) for a basic morphological evaluation. Images were acquired under a Nikon E600 optical microscope. For skeletal muscle morphology, biopsies from the gastrocnemius muscle were collected and snap frozen in isopentane pre-cooled in liquid nitrogen. All specimens were frozen for a maximum of 2 h after sampling. Frozen sections (10 µm thick) of skeletal muscle were subjected to a standard panel of histochemical staining, including hematoxylin and eosin (H&E), Engel’s trichrome (ET), NADH-tetrazolium reductase (NADH-TR), succinate dehydrogenase (SDH), cytochrome oxidase (COX), ATPase at pH 10.4, non-specific esterase (NSE), and periodic acid–Schiff (PAS) [29]. 

The following scoring system was used to assess the degree of fiber atrophy (mild, moderate, and severe):

- <10% of atrophic fibers/100 fibers at 20× magnification (mild)
- 10–50% of atrophic fibers/100 fibers at 20× magnification (moderate)
- >50% of atrophic fibers/100 fibers at 20× magnification (severe)

At least five fields at 40× magnification were evaluated for each section by two independent pathologists (O.P. and D.D.B.) under a Nikon E600 optical microscope, with a concordance rate of 95%.

To assess skeletal muscle development from E17.5 embryo mice, diaphragm thickness measurements were taken from liver-level cross sections as previously described [30]. Digitized muscle images were taken with an optical microscope (Nikon E600; Nikon, Tokyo, Japan) equipped with a digital camera (Nikon DMX1200). The size and diameter of individual cells were determined by the lines across the diameter of each muscle fiber as previously described [30]. Multiple samples (20 cells per sample) were analyzed.

Wheat germ agglutinin (WGA) Alexa Fluor 488 conjugate staining was performed on cardiac sections of mice at different ages (4, 12, and 18 months). Mouse heart specimens were fixed in 4% formaldehyde and embedded in paraffin. After deparaffinization and rehydration, 4 μm thick sections were prepared and mounted on glass slides. An even number of cardiac cross sections per group were stained with WGA; nuclei were detected by 4′-6-diamidino-2-phenylindole (DAPI). Slides were analyzed with a fluorescence microscope and analyzed using a Nikon light microscope, and the cardiomyocyte area was measured using a computer-assisted image analysis software (ImageJ software, National Institutes of Health; Bethesda, MD, USA,). For the assessment of the cardiomyocyte cross-sectional area, the mean area was evaluated by measuring 200–400 cells per heart (*n* = 2–4 animals/group). Fibrotic regions (6–8 fields/section, *n* = 2–4 animals/group) were measured as percentage of collagen-stained area/total myocardial area and averaged using ImageJ software. 

### 4.3. Immunohistochemistry

To evaluate the expression of myogenin in E17.5 mice, 4 μm sections were deparaffinized in xylene and rehydrated in a decreasing series of alcohol. Peroxidases were blocked with a solution of hydrogen peroxide and methanol (4:1) for 15 min. Antigen retrieval pretreatments were performed using a HIER citrate buffer, pH 6.0 (Bio-Optica, Milan, Italy) for 20 min at 98 °C. We used a mouse monoclonal antibody anti-myogenin (sc-12732; Santa Cruz Biotechnology, Dallas, TX, USA) at 1:50 in PBS. The antibody has a mouse host; for this reason, immunohistochemistry was performed according to the protocol indicated by the M.O.M. Kit (Cat. No. PK-2200, Vector Laboratories, Burlingame, CA, USA) [31].

The DAB stain was quantified with FIJI ImageJ. For each case, three random 40× fields of the transverse section of the limb skeletal muscle were photographed under an optical microscope (Nikon E600; Nikon, Tokyo, Japan) associated with a digital camera (Nikon DMX1200).

### 4.4. RNA Extraction and Real-Time PCR

Total RNA from the left ventricle of frozen heart samples obtained from 12-month-old murine hearts (WT, *n* = 3 and *Hipk2*-KO, *n* = 3) was extracted using TRIzol reagent (Invitrogen) [32]. cDNA was synthesized from total RNA through reverse transcription, and subsequent qRT-PCR analysis was performed using the Power SYBR Green PCR Master Mix (Applied Biosystems, Foster City, CA, USA) as previously described [33]. 

The following primers were used:

- mouse-NPPA. Fw: 5′-CACAGATCTGATGGATTTCAAGA-3′
- mouse-NPPA. Re: 5′-CCTCATCTTCTACCGGCATC-3′
- mouse-NPPB. Fw: 5′-GTCAGTCGTTTGGGCTGTAAC-3′
- mouse-NPPB. Re: 5′-AGACCCAGGCAGAGTCAGAA-3′
- mouse-MYH7. Fw: 5′-CGGAAACTGAAAACGGAAAG-3′
- mouse-MYH7. Re: 5′-TCCTCGATCTTGTCGAACTTG-3′
- mouse-Rps18. Fw: 5′-AAACGGCTACCACATCCAAG-3′
- mouse-Rps18. Re: 5′-CCTCCAATGGATCCTCGTTAA-3′

### 4.5. Statistical Analysis

Data are expressed as mean ± standard deviation (SD). Statistical significance between groups was assessed by Student’s *t*-test. For all analyses, a minimum value of *p* < 0.05 was considered significant.

## 5. Conclusions

In conclusion, the data presented here contribute to define the physiological role of *Hipk2*, which, considering the late onset of all the phenotypic anomalies displayed by KO mice, could be particularly important during the process of aging. Moreover, our data suggest the possible involvement of HIPK2 in the pathogenesis of multiple neurological, muscular, and cardiac diseases. 

## Supplementary Materials

The following are available online at https://www.mdpi.com/article/10.3390/ijms22158294/s1.
