# Supplementary material for: Phenotypic Effects of Homeodomain-Interacting Protein Kinase 2 Deletion in Mice
Source: Int J Mol Sci. 2021 Aug 2;22(15):8294. doi: 10.3390/ijms22158294 (PMC8348407; doi:10.3390/ijms22158294)
Supplement: Supplementary file 1 [file ijms-22-08294-s001.zip › ijms-1321565-supplementary.pdf]

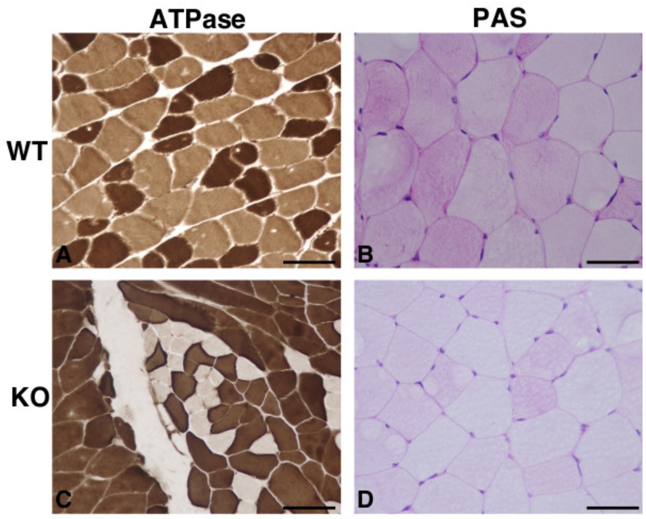

Figure S1. Analysis of fiber type grouping and glycogen accumulation in *Hipk2*-KO skeletal muscle. ATPase (**A**,**C**) and PAS (**B**,**D**) staining from sections of skeletal muscle of 18-month-old WT and *Hipk2*-KO mice are shown. Original magnification, 40x. One representative experiment is shown. Scale bar = 20 μm.

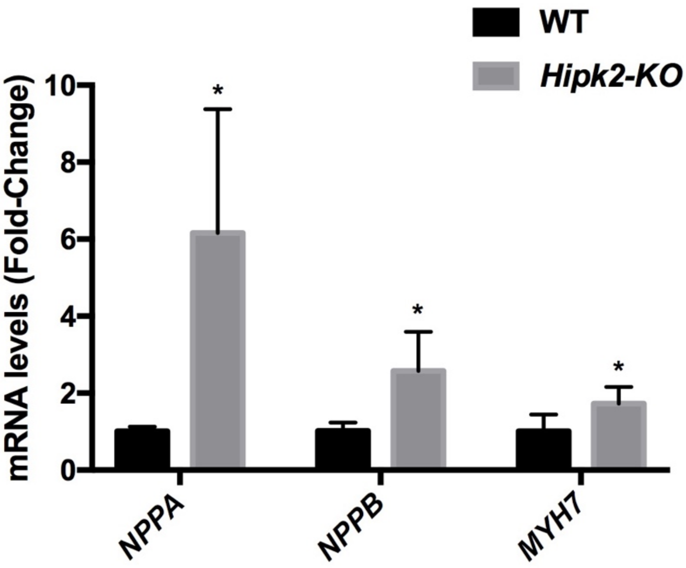

Figure S2. Analysis of expression level of heart failure markers in WT and *Hipk2*-KO mice. RNA extracted from hearts of 12-month-old WT and *Hipk2*-KO mice were analyzed by qRT-PCR for *NPPA, NPPB* and *MYH7* expression. The *RPS18* expression level has been used for normalization. Data are mean ± standard deviation (SD) of a representative experiment performed in triplicate (*p<0.05).
